# Efficacy and safety of caspofungin for patients with hepatic insufficiency

**DOI:** 10.1186/s12879-022-07527-8

**Published:** 2022-06-20

**Authors:** Xiaoyun Ran, Pengfei Wang, An Zhang, Binfei Tang

**Affiliations:** Department of Critical Care Medicine, The Second Affiliated Hospital of Chongqing Medical University, Chongqing, 400010 China

**Keywords:** Hepatic insufficiency, Child–Pugh score, Caspofungin, Dosage, Efficacy

## Abstract

**Background:**

To observe the changes of hepatic function and efficacy of conventional dosage of caspofungin in the treatment of patients with different Child–Pugh scores.

**Methods:**

In total, 200 patients (Child–Pugh A group: 66 patients, Child–Pugh B group: 83 patients, Child–Pugh C group: 51 patients) treated with caspofungin from May 2018 to March 2021 in the Second Affiliated Hospital of Chongqing Medical University were enrolled. Main investigation items were as follows: sex, age, weight, duration of treatment, dosage, department, underlying diseases, risk factors for fungal infection, albumin, liver enzyme, total bilirubin, serum creatinine, estimated glomerular filtration rate. To investigate the changes of liver, kidney function tests and efficacy during the treatments of caspofungin. Patients were divided into three groups according to the duration of treatment of caspofungin:1-week group, 2-week group and 3-week group, respectively.

**Results:**

In the three groups, albumin, liver enzyme levels, total bilirubin and serum creatinine, estimated glomerular filtration rate had no significant difference (P > 0.05). The efficacy of different Child–Pugh scores and different duration of treatment was also significantly different (P > 0.05).

**Conclusions:**

Caspofungin is well tolerated and highly effective. And it will not exacerbate the hepatic and renal function when administered with the not-reducing dose, which indicate the clinical application value of caspofungin. Besides, extending the treatment duration has little effect on improving the efficacy of caspofungin. The drug should be withdrawn timely according to the patients’ clinical condition in order to reduce the adverse reactions and economic burden.

## Background

Since the start of the new millennium, invasive fungal infection (IFI) has drastically increased. IFI related mortality rates is 27.6%, there are about 100, 000 in-patients with IFI every year, and the annual cost of treating IFI in the United States is more than $7 billion [1]. Therefore, the selection of appropriate and effective antifungal drugs is an important factor to alleviate morbidity and economic burden of patients.

Currently available antifungal agents for IFIs includes echinocandins, polyenes, flucytosine, triazoles. But, a series of adverse drug reactions (ADRs) were followed with the widespread use of antifungal drugs. The most common ADRs are hepatotoxicity, nephrotoxicity and hypokalemia [2–4].

Caspofungin, as the representative of echinocandins, is generally well tolerated and safety [5, 6]. The most common abnormal laboratory index about caspofungin is elevation of liver function values, manifested by increased serum alkaline phosphatase and transaminase concentrations, and the increase of serum creatinine and blood urea nitrogen [7, 8].

However, the research on the application of caspofungin in patients with hepatic insufficiency (HI) is insufficient. In order to guide the clinical diagnosis and treatment of antifungal drugs in patients with HI. This study collected relevant clinical cases, revealed the clinical effect of caspofungin in patients with HI, and analyzed the changes of laboratory indexes such as liver and kidney function in patients treated with caspofungin. The report is as follows.

## Materials and methods

### Inclusion criteria and study design

This study was a retrospective single-center analysis, designed to estimate the changes of hepatic function and efficacy of caspofungin (Cancidas^®^, Merck & Co. Inc., Kajing^®^, Jiangsu Hengrui Medicine Co. Ltd) used for the confirmed, clinically diagnosed and suspected of IFI in the Second Affiliated Hospital of Chongqing Medical University during May 2018 to March 2021. Clinical profiles and laboratory parameters of the patients, were evaluated. All patients aged > 18 years, treatment duration ≥ 7 days, matched with *The Chinese guidelines for the diagnosis and treatment of invasive fungal disease in patients with hematological disorders and cancers (the 6th revision), Guidelines for the diagnosis and treatment of Invasive fungal infection in critical ill patients (2007)* were included in the study.

The Child–Pugh score was graded as 5–6 points for Child–Pugh A, 7–9 points for Child–Pugh B, and 10–15 points for Child–Pugh C. Patients were be divided into mild, moderate, or severe by the corresponding Child–Pugh score A, B and C.

The standard dose of caspofungin to treat IFI was a 70-mg loading dose followed by a once-daily maintenance dose of 50 mg infused over 1 h. All patients were administered with caspofungin. Efficacy was assessed in all patients at the end of caspofungin therapy and the hepatic and renal functions were recorded before administration (D0), the first day (D1), the 7th day (D7), the 14th day (D14), the 21th day (D21) and the 28th day (D28) of the administration, which included the albumin, alamine aminotransferase (ALT), aspartate aminotransferase (AST), alkaline phosphatase (ALP), γ-glutamyl transpeptidase (GGT), total bilirubin (TBIL) and serum creatinine (Scr), estimated glomerular filtration rate (eGFR).

Criterias for efficacy was referred to the *Mycoses Study Group and European Organization for Research and Treatment of Cancer Consensus Criteria (2008)* [9], and the efficacy was defined as complete response, partial response, stable response, progression of fungal disease, death.

### Statistical analysis

Statistical analysis was performed through IBM SPSS Statistics 21. The enumeration data were expressed as percentage (%), and the measurement data as median (quartile) (M, P25-P75), Friedman test was used to compare the changes of various parameters in different times during medication, and Wilcoxon signed-rank test was used to compare the differences between the two groups. In order to avoid Type I error caused by pairwise comparison of multiple samples, Bonferroni's correction was needed, P values < 0.05 were regarded as statistically significant. Chi-square test was used to compare the efficacy, P values < 0.05 indicated that the difference was statistically significant.

## Results

### Patient characteristics

Characteristics of the 200 patients evaluated in the study were shown in Table 1. Fifty-four (27%) patients had suffered from hematologic malignancies, fifty-one (25.5%) patients had liver cirrhosis, followed by severe pulmonary diseases (18.5%) and malignancies (10%). Pulmonary invasive fungal infection is the most common, with a total of 69.5%, followed by digestive tract (24%), blood (3.5%), urinary tract (1%).Six (3%) patients with confirmed IFI were administered caspofungin as primary therapy. One hundred and fourteen (57%) with clinical diagnosis, seventy-four (37%) with suspected diagnosis patients were administered caspofungin empirically.

**Table 1:** General characteristics of patients

Characteristic		Child–Pugh classification			Total (N = 200, %)	P	Statistic (X^2^/F)
		A (n = 66)	B (n = 83)	C (n = 51)			
Sex	Female	36	48	36	120 (60)	0.186	3.363
Age	M (P25-P75)	58 (42–72)	59 (48–68)	49 (43–61)	–	0.015	4.319
Weight	M (P25-P75)	56.6 (50–66)	58.6 (50–65)	58.6 (54–65)	–	0.376	0.984
Period of treatment	1 week	26	36	17	79 (39.5)	0.514	1.333
	2 weeks	22	28	22	72 (36)	0.469	1.516
	3 weeks	18	19	12	49 (24.5)	0.812	0.416
Department	Infectious Diseases	6	24	44	74 (37)	0.000	77.503
	Hematology	36	20	1	57 (28.5)	0.000	40.389
	ICU	2	13	1	16 (8)	0.003	11.364
	Respiratory	4	12	4	20 (10)	0.198	3.234
	Nephrology	3	2	0	5 (2.5)	0.375	2.091
	Rheumatology and Immunology	2	2	0	4 (2)	0.683	1.310
	Others	13	10	1	24 (12)	0.014	8.570
Underlying disease	Cirrhosis of the liver	0	10	41	51 (25.5)	0.000	111.386
	Haematological malignancy	34	19	1	54 (27)	0.000	37.058
	Severe pulmonary disease	11	24	2	37 (18.5)	0.001	13.308
	Cancer	6	14	0	20 (10)	0.008	10.077
	Sepsis	1	0	5	6 (3)	0.003	8.708
	Acute pancreatitis	1	3	1	5 (2.5)	0.853	0.719
	Solid organ transplantation	4	0	0	4 (2)	0.015	5.941
	Autoimmune disease	3	2	0	5 (2.5)	0.375	2.091
	Chronic kidney disease	2	1	0	3 (1.5)	0.616	1.509
	Atherosclerotic vascular disease	2	1	0	3 (1.5)	0.616	1.509
	Others	2	9	1	12 (6)	0.077	5.103
Risk-factor for fungal infection	Broad-spectrum antibiotic	64	81	51	196 (98)	0.683	1.310
	Corticosteroid	39	42	2	83 (41.5)	0.000	40.910
	Immunosuppression	25	19	2	46 (23)	0.000	18.733
	Central venous line	31	42	33	106 (53)	0.142	3.960
	Recent surgery	5	12	1	18 (9)	0.041	6.268
	Tracheal intubation	8	31	6	45 (22.5)	0.000	17.943
	Malignancy	6	14	2	22 (11)	0.055	5.775
	Diabete	12	12	4	28 (14)	0.285	2.579
	Transplant recipient	4	0	0	4 (4)	0.015	5.941
	HIV	0	2	1	3 (1.5)	0.481	1.560
Hepatoprotective drugs	Yes	35	60	48	143 (71.5)	0.000	18.074
Diagnostic grades of IFI Infection site	Confirmed	2	8	2	12 (6)	0.023	3.319
	Clinical diagnosis	31	47	36	114 (57)	0.038	6.556
	Suspected diagnosis	33	28	13	74 (37)	0.018	8.063
	Pulmonary	54	58	27	139 (69.5)	0.003	12.327
	Digestive tract	10	16	22	48 (24)	0.001	14.088
	Blood	1	5	1	7 (3.5)	0.260	2.693
	Urinary tract	0	1	1	2 (1)	0.725	1.408
	Others	1	3	0	4 (2)	0.462	1.681

### Dose and duration of treatment

The mean duration of caspofungin treatment was 16.8 days (range 7–62 days). Caspofungin therapy was started at a dose of 70 mg followed by 50 mg/day in 166 (83%) patients. Twenty-six (13%) patients received a 50 mg maintenance dose of caspofungin daily. Eight (4%) received caspofungin 50 mg/day, following a loading dose of 100 mg on day 1.

### Changes of liver and kidney function

During the treatment, the doctor would withdraw caspofungin according to the general condition, laboratory examination parameters, imaging examinations or economic reasons of the patients. Therefore, based on the treatment duration, patients were divided into 1-week group, 2-week group and 3-week group (Table 1).

The Changes of liver and kidney function of 1-week group were shown in Table 2. ALP, GGT and Scr in Child–Pugh A patients and GGT in Child–Pugh C patients changed significantly during the treatment. But when making a pairwise comparison of different time points, we found that only GGT in Child–Pugh A patients on D0 significantly larger than D1 and D7 (P < 0.05), GGT in Child–Pugh A patients on D1 significantly larger than D7 (P < 0.05). The results showed that the liver and kidney function in Child–Pugh A, B and C patients did not changed significantly with time (P > 0.05).

**Table 2:** Comparison of the changes of liver and kidney function in patients with different Child–Pugh scores in the 1 week group (M (P25–P75))

Group	Time	albumin g/L	ALT U/L	AST U/L	ALP U/L	GGT U/L	TBIL U/L	Scr mmol/L	eGFR ml/min
A	D0 D1 D7 X2 *P*	33.7 (29–37) 33.3 (30–35) 31.4 (29–33) 5.79 0.06	28.0 (13–60) 24.5 (12–71) 21.5 (12–41) 2.80 0.25	20.0 (16–40) 20.5 (13–38) 22.5 (13–40.) 3.98 0.14	85.0 (72–133) 85.5 (60–124) 80.0 (63–108) 9.41 0.01	68.0 (31–191) 67.0 (29–172)^*^ 52.0 (22–100)^*#^ 14.00 0.01	9.0 (5–13) 8.8 (5–12) 7.9 (6–11) 1.17 0.56	62.9 (53–85) 60.9 (48–86) 61.0 (45–71) 6.39 0.04	78.8 (52–96) 87.7 (60–107) 94.9 (63–120) 5.43 0.06
B	D0 D1 D7 X^2^ *P*	30.0 (28–32) 29.3 (26–32) 29.9 (28–32) 1.69 0.43	37.5 (16–80) 36.0 (12–71) 27.5 (14–59) 1.922 0.382	48.5 (27–84) 45.5 (23–99) 60.1 (25–86) 0.75 0.69	115.5 (68–147) 114.5 (70–154) 112.0 (90–209) 0.14 0.93	83.0 (29–165) 88.0 (34–153) 92.5 (37–143) 2.02 0.37	21.1 (10–42) 13.8 (8–40) 18.2 (8–36) 3.32 0.21	68.7 (56–100) 77.0 (50–106) 81.8 (58–112) 0.39 0.82	81.6 (46–106) 79.4 (45`104) 77.4 (37–98) 0.2 0.91
C	D0 D1 D7 X2 *P*	30.6 (25–34) 30.0 (27–35) 31.0 (27–34) 0.22 0.89	21.0 (16–32) 22.0 (14–32) 17.0 (10–41) 0.456 0.796	48.0 (28–85) 44.0 (30–65) 55.0 (33–117) 2.63 0.27	88.0 (71–171) 77.0 (67–157) 86.0 (68–153) 5.52 0.06	37.0 (25–85) 37.0 (24–60) 46.0 (23–66) 7.18 0.03	163.0 (51–324) 156.0 (67–276) 162.0 (62–308) 0.65 0.72	89.0 (54–146) 83.4 (62–168) 79.9 (64–142) 0.78 0.68	69.6 (30–91) 69.7 (32–97) 65.0 (36–86) 1.00 0.61

*Bonferroni's correction, compared with D0, the difference was statistically significant

^#^Bonferroni's correction, compared with D1, the difference was statistically significant

In the 2-week group (Table 3), the albumin, ALT, AST and Scr, eGFR in Child–Pugh B patients and ALT in Child–Pugh C patients changed significantly with the time. But when making a pairwise comparison of different time points, we found that the albumin levels in Child–Pugh B patients on D1 were significantly less than D14 (P < 0.05),Scr in Child–Pugh B patients on D0 and D1 significantly larger than D7 and D14, eGFR in Child–Pugh B patients on D0 and D1 significantly less than D7 and D14, respectively (P < 0.05). The results showed that the liver and kidney function in Child–Pugh A, B and C patients had not changed significantly with time (P > 0.05).

**Table 3:** Comparison of the changes of liver and kidney function in patients with different Child–Pugh scores in the 2-week group (M (P25–P75))

Group	Time	Albumin g/L	ALT U/L	AST U/L	ALP U/L	GGT U/L	TBIL U/L	Scr mmol/L	eGFR mL/min
A	D0 D1 D7 D14 X^2^ *P*	31.8 (29–35) 32.4 (29–34) 30.1 (26–33) 31.6 (29–37) 6.39 0.09	17.5 (8–35) 16.0 (9–31) 25.5 (14–48) 19.5 (12–36) 3.38 0.29	19.5 (16–28) 16.5 (13–26) 20.0 (15–26) 22.0 (12–35) 4.91 0.18	89.0 (59–113) 86.0 (74–118) 94.7 (81–103) 96.0 (64–128) 1.41 0.70	38.0 (19–92) 42.0 (27–84) 38.5 (26–61) 36.0 (22–82) 2.08 0.56	8.6 (6–15) 8.8 (5.5–14) 10.7 (6–15) 11.1 (9–19) 2.35 0.50	56.4 (47–131) 57.4 (45–108) 69.0 (44–115) 65.5 (42–126) 0.60 0.89	86.8 (38–130) 88.6 (38–139) 88.4 (41–131) 85.9 (31–145) 0.96 0.81
B	D0 D1 D7 D14 X^2^ *P*	28.1 (26–31) 27.6 (25–29) 29.5 (27–31) 30.1 (27–34)^#^ 13.03 0.005	26.0 (14–68) 27.0 (12–50) 28.0 (10–41) 20.5 (11–37) 9.24 0.03	42.5 (23–56) 44.5 (24–59) 38.0 (25–61) 32.0 (20–52) 9.92 0.03	110.0 (71–174) 116.0 (72–175) 115.5 (74–182) 134.0 (69–186) 1.14 0.77	79.5 (46–129) 82.5 (49–134) 79.0 (49–123) 63.5 (41–86) 5.04 0.17	20.5 (10–52) 17.4 (13–67) 25.1 (10–55) 18.6 (9–48) 4.99 0.17	77.7 (48–112) 65.7 (48–98) 49.5 (37–80)^*#^ 58.3 (39–80)^*#^ 28.05 0.00	85.1 (42–105) 81.4 (48–100) 103.9 (65–134) ^*#^ 105.4 (59–138) ^*#^ 22.6 0.00
C	D0 D1 D7 D14 X^2^ *P*	29.1 (26–32) 30.1 (27–33) 30.7 (28–33) 31.8 (27–34) 2.29 0.51	49.5 (35–91) 50.0 (26–110) 45.0 (22–73) 37.0 (21–73) 9.65 0.02	76.0 (56–123) 73.0 (53–113) 81.5 (49–117) 71.0 (42–98) 2.18 0.54	135.0 (100–164) 132.0 (110–171) 117.0 (73–150) 115.0 (93–150) 6.08 0.11	51.0 (30–81) 51.0 (28–91) 56.0 (34–80) 46.0 (30–74) 0.19 0.76	240.9 (110–371) 263.1 (88–388) 227.3 (109–335) 196.0 (64–332) 3.61 0.31	71.3 (52–101) 65.5 (43–80) 61.4 (41–77) 68.9 (54–91) 1.33 0.72	102.9 (59–136) 106.9 (71–144) 110.5 (59–139) 104.8 (60–130) 0.88 0.83

^*^Bonferroni's correction, compared with D0, the difference was statistically significant

^#^Bonferroni's correction, compared with D1, the difference was statistically significant

In the 3-week group (Table 4), the albumin and Scr, eGFR levels in Child–Pugh B patients changed significantly with time. But when making a pairwise comparison of different time points, we found that albumin levels in Child–Pugh B patients on D1 were significantly less than D14 and D21 (P < 0.05), Scr levels in Child–Pugh B patients on D0 were significantly larger than D1 (P < 0.05), eGFR levels in Child–Pugh B patients on D0 and D1 significantly less than D14 and D21. The results showed that the liver and kidney function in Child–Pugh A, B and C patients had not changed significantly with time (P > 0.05).

**Table 4:** Comparison of the changes of liver and kidney function in patients with different Child–Pugh scores in the 3-week group (M (P25–P75))

Group	Time	Albumin g/L	ALT U/L	AST U/L	ALP U/L	GGT U/L	TBIL U/L	Scr mmol/L	eGFR mL/min
A	D0 D1 D7 D14 D21 X^2^ *P*	31.7 (28–35) 29.5 (27–32) 30.1 (28–32) 30.2 (29–32) 29.5 (29–35) 4.57 0.33	19.0 (8–28) 15.0 (10–43) 16.0 (9–26) 20.0 (9–31) 21.0 (9–38) 0.65 0.96	18.0 (11–25) 19.0 (11–28) 21.0 (13–28) 20.0 (12–28) 27.0 (13–34) 1.48 0.83	72.0 (55–109) 81.0 (58–122) 84.0 (59–114) 82.0 (61–124) 76.0 (67–123) 4.15 0.48	45.0 (23–119) 51.0 (18–129) 36.0 (19–101) 39.0 (26–81) 39.0 (25–56) 1.71 0.79	7.8 (5–18) 7.2 (5–17) 7.6 (5–15) 8.3 (6–13) 8.2 (6–12) 1.21 0.87	78.2 (55–105) 65.7 (54–100) 67.1 (57–108) 62.5 (51–90) 54.7 (45–86) 7.51 0.11	85.5 (31–152) 93.4 (41–142) 83.5 (27–125) 94.0 (37–145) 95.6 (36–146) 8.08 0.08
B	D0 D1 D7 D14 D21 X^2^ *P*	28.0 (26–32) 27.1 (26–28) 30.1 (29–32) 31.6 (29–34)^#^ 31.1 (29–33)^#^ 17.26 0.002	15.0 (11–55) 22.0 (8–57) 17.0 (11–48) 25.0 (9–56) 23.5 (13–47) 1.65 0.79	24.0 (14–50) 24.0 (13–76) 22.0 (12–48) 23.0 (10–52) 22.0 (16–32) 3.07 0.45	97.0 (63–140) 106.0 (67–137) 118.0 (71–176) 103.0 (72–201) 86.0 (70–208) 4.61 0.33	67.0 (32–181) 63.1 (26–166) 48.0 (20–178) 87.0 (19–133) 72.0 (23–169) 2.19 0.70	7.7 (6–11) 9.0 (4–12) 9.3 (6–11) 10.6 (8–16) 9.0 (6.1–14) 4.60 0.33	67.9 (51–118) 62.2 (48–118)^*^ 58.9 (45–94) 52.7 (23–71) 53.5 (41–76) 12.54 0.01	70.5 (38–110) 81.4 (42–125) 90.9 (56–133) 99.6 (43–132) ^*#^ 106.1 (37–135) ^*#^ 11.1 0.02
C	D0 D1 D7 D14 D21 X^2^ *P*	29.4 (28–31) 29.6 (29–31) 30.5 (30–32) 31.0 (30–32) 33.0 (32–35) 9.08 0.06	44.0 (16–71) 26.0 (10–52) 18.0 (11–41) 22.0 (11–41) 20.0 (15–40) 1.93 0.75	56.0 (44–159) 56.0 (33–106) 55.0 (41–92) 47.0 (33–63) 55.0 (34–84) 2.94 0.57	145.0 (99–163) 130.0 (116–155) 122.0 (91–157) 121.0 (112–160) 123.0 (92–189) 3.34 0.50	54.0 (30–82) 51.0 (30–68) 41.0 (24–87) 47.0 (31–68) 42.0 (25–70) 1.72 0.79	310.6 (121–400) 276.5 (121–417) 284.5 (110–393) 184.0 (119–358) 171.4 (121–407) 5.49 0.24	60.5 (47–386) 60.5 (54–199) 68.1 (43–114) 53.5 (45–87) 60.1 (42–75) 3.36 0.49	77.8 (14–128) 69.8 (3–128) 96.8 (53–147) 87.4 (47–130) 93.8 (61–116) 3.58 0.46

^*^Bonferroni's correction, compared with D0, the difference was statistically significant

^#^Bonferroni's correction, compared with D1, the difference was statistically significant

### The outcomes of treatment

At the end of treatment, efficacy with different Child–Pugh scores and different courses of treatment was 63%. There was no difference in the effective rate of patients classified as Child–Pugh A, B and C (P > 0.05). There was no difference in the effective rate of patients with 1 week, 2 weeks and 3 weeks of treatment (P > 0.05) (Tables 5, 6).

**Table 5:** Comparison of the efficacy in different Child–Pugh scores patients

Group	Complete response	Partial response	Stable response	Progression of disease	Death	Efficient (%)
A B C X2 P Total	2 3 5 2.939 0.245 10	46 48 22 8.333 0.016 116	8 9 11 3.307 0.212 28	7 9 6 0.043 1.000 22	3 14 7 5.479 0.067 24	72.7 61.4 52.9 7.438 0.114 63.0

**Table 6:** Comparison of the efficacy in different treatment duration

Group	Complete response	Partial response	Stable response	Progression of disease	Death	Efficient (%)
1 week 2 weeks 3 weeks X2 P	4 5 1 1.269 0.652	46 40 30 6.998 0.030	12 10 5 1.985 0.369	9 8 6 0.700 0.715	8 9 8 0.690 0.711	63.3 62.5 63.3 1.416 0.084
Total	10	116	27	22	25	63.0

## Discussion

The recent literature suggests that common adverse effects of caspofungin include elevated transaminases (ALT, AST), ALP, TBIL, Scr, fever, GI symptoms (nauseating, vomiting, abdominal pain, diarrhea), phlebitis, and allergy. In HI patients, the dose should be adjusted according to Child–Pugh score. In recent years, Gustot et al. [10] found that the dose of caspofungin should not be reduced regardless of the severity of hepatic failure. In the present study, 13% patients were not given loading doses for economic reasons, pre-existing use of other antifungal drugs, or irrational dosing, but all patients (including Child–Pugh C patients) were maintained at 50 mg/day regardless of hepatic function, and no exacerbation of hepatic or renal impairment occurred regardless of the duration. One one hand, it may be related to the aggressive treatments in primary diseases, which avoided mild hepatic impairment in some patients. On the other hand, some patients were treated with hepatoprotective drugs during hospitalization for avoiding the underlying hepatic impairment [8]. Besides, in this study there were differences in the basic liver conditions between the groups, for example, 80% of patients with liver cirrhosis in grade C group, but with the use of hepatoprotective drugs, the findings suggest that the use of standard doses of caspofungin is still safe and no adjustment of dose, while the percentage of cirrhosis in the grade B group was only 12 and the use of liver-protective drugs in this subgroup was 72%, suggesting that standard-dose caspofungin remains tolerable and safe through the use of liver-protective drugs in patients with non-cirrhosis leading to abnormal liver function and graded at grade B. Therefore, our study indicates that caspofungin is safe and reliable for using in IFI patients, with minimal effect on liver function. If patients with basic HI, we should pay attention to monitoring their liver function and adding hepatoprotective drugs in time while not discontinuation of the drug.

At present, many scholars at home and abroad have conducted studies on high loading doses or high maintenance doses of caspofungin in order to further explore the maximum tolerated dose and efficacy of caspofungin. Wang Huajie et al. [11] concluded that the high dose (a loading dose of 100 mg on day 1 and maintenance dose of 70 mg/day) caspofungin group had significantly higher clearance rate of different types of fungi compared with the standard dose group, and there were no significant changes in liver and kidney function before and after treatment in both groups. In this study, due to physician decisions and the patient's illness, 8 (4%) patients (all from the infection department, 7 with underlying liver failure and 1 with severe pulmonary infection) were administered with a loading dose of 100 mg on day 1 and a maintenance dose of 50 mg/day for 7–24 days, and 2 of these patients eventually died without further deterioration in liver function during treatment, which is consistent with the study carried by Wang, Huajie et al. It indicates that increasing the first dose of caspofungin does not aggravate patients’ hepatic impairment, However, because the sample size is small, the outcomes for these patients should be interpreted with caution. But tit can still provide a reference for future studies.

In terms of efficacy, the overall effective rate (63%) when treating with caspofungin in patients with different Child–Pugh classifications was almost consistent with the effective rate (65%) reported by Xiaohui Zhang et al. [12]. There was no difference in the efficacy of caspofungin in patients with different hepatic function grades, suggesting that even without dose reduction, grade B and C patients tolerated caspofungin not differently from grade A patients and had better treatment outcomes. The current consensus on the time frame of antifungal therapy [13] revealed that antifungal drugs should be maintained at least 2 weeks after the patient's signs and symptoms have alleviated, laboratory parameters have improved, and microbial detection has turned negative. However, this study showed that there was no difference in the efficiency of caspofungin in the 1-week, 2-week, and 3-week antifungal treatment, indicating that patients' symptoms and signs, laboratory indices, imaging, and pathogenesis should be followed up timely and antifungal treatment should be withdrawn according to their clinical conditions timely. Because blindly prolonging the duration of caspofungin treatment if the patients' above monitoring indices have improved significantly does not seem to improve the patients' outcomes significantly but increase their financial burden.

## Conclusions

Based on these limited data, it is suggested that caspofungin is well tolerated and liver function classified as Child–Pugh C should not be considered as a contraindication for caspofungin using or a criterion for dose reduction, and caspofungin should be administered in adequate doses even in HI patients to achieve better therapeutic outcomes.

## Data Availability

The datasets used during the current study are available from the corresponding author upon reasonable request.
